# Supplementary material for: Distinctive Kaposi Sarcoma-Associated Herpesvirus Serological Profile during Acute Plasmodium falciparum Malaria Episodes
Source: Int J Mol Sci. 2023 Apr 4;24(7):6711. doi: 10.3390/ijms24076711 (PMC10095526; doi:10.3390/ijms24076711)
Supplement: Supplementary file 1 [file ijms-24-06711-s001.zip › ijms-2230315-supplemantary figure.pdf]

Supplementary Figure

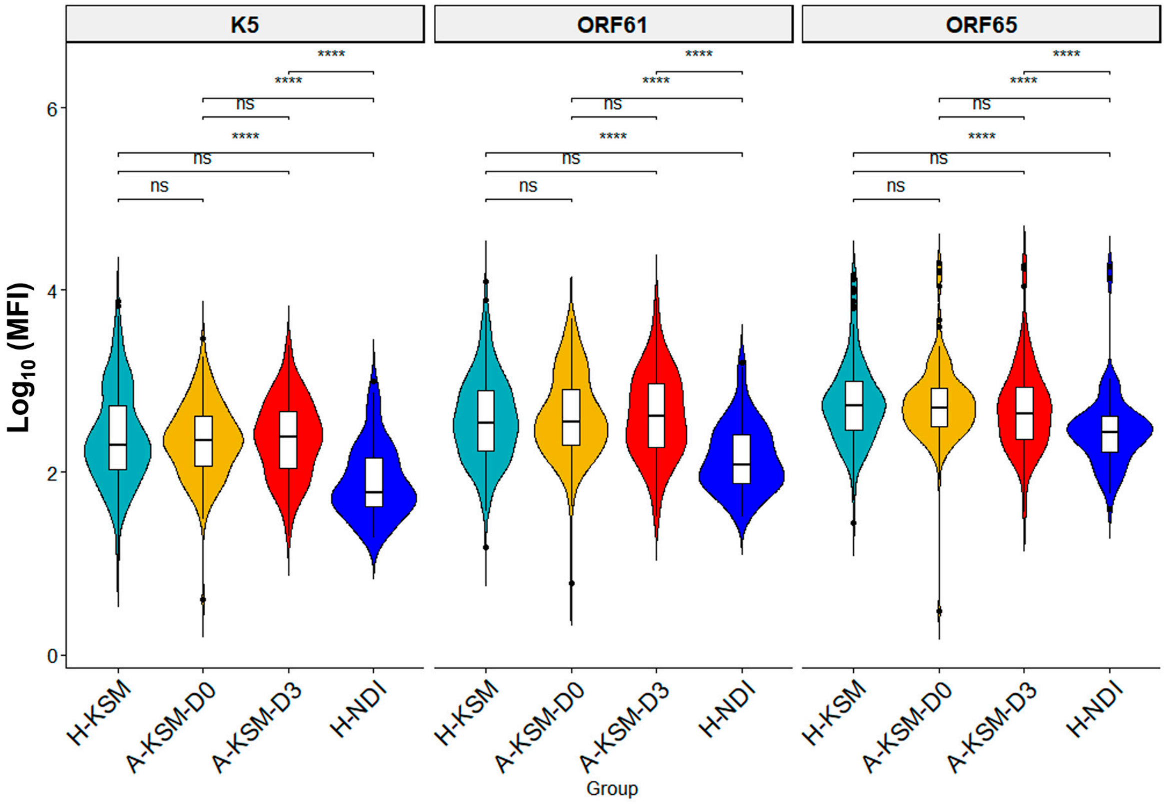

Figure S1. Additional comparison of IgG antibodies against K5, ORF61 and ORF65 antigens in A-KSM, H-KSM and H-NDI children.) (**** $p < 0.0001$, ns: $p > 0.05$).

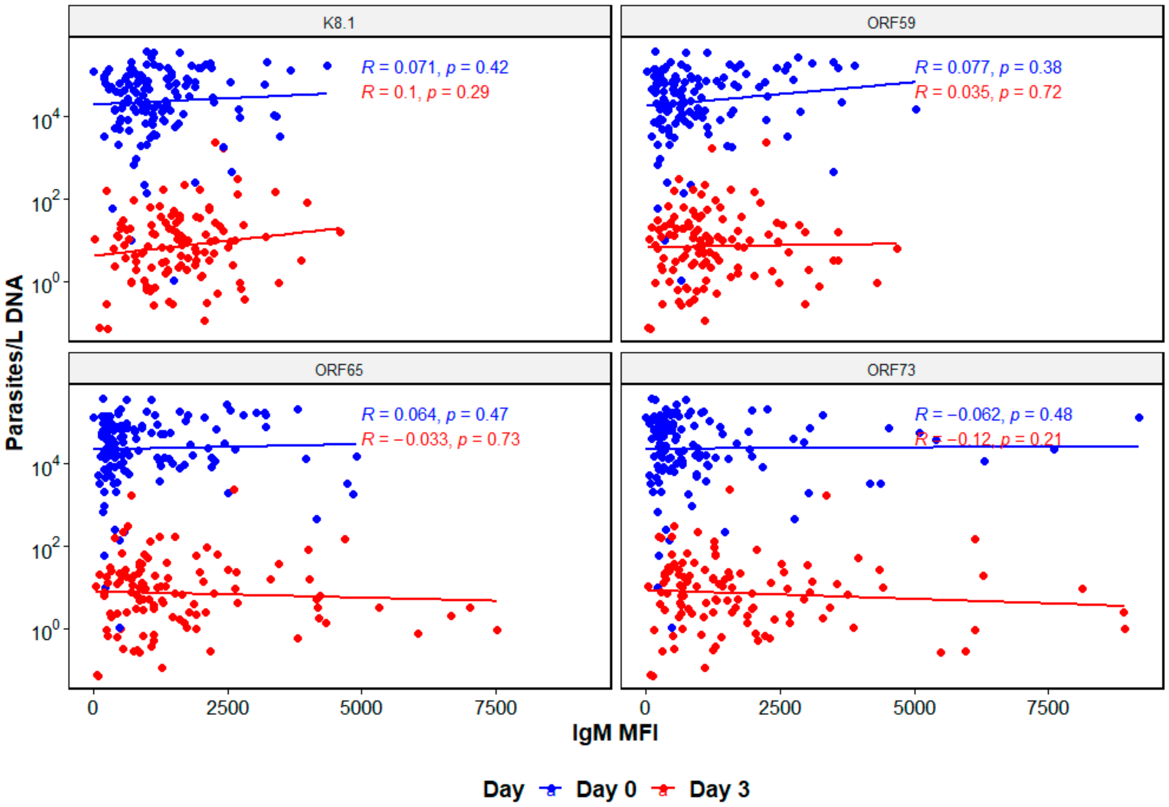

Figure S2. Correlation between anti-KSHV IgM antibodies (K8.1 ORF59, ORF65 and ORF73) with *P. falciparum* malaria parasite density in A-KSM-D0 and A-KSM-D3.

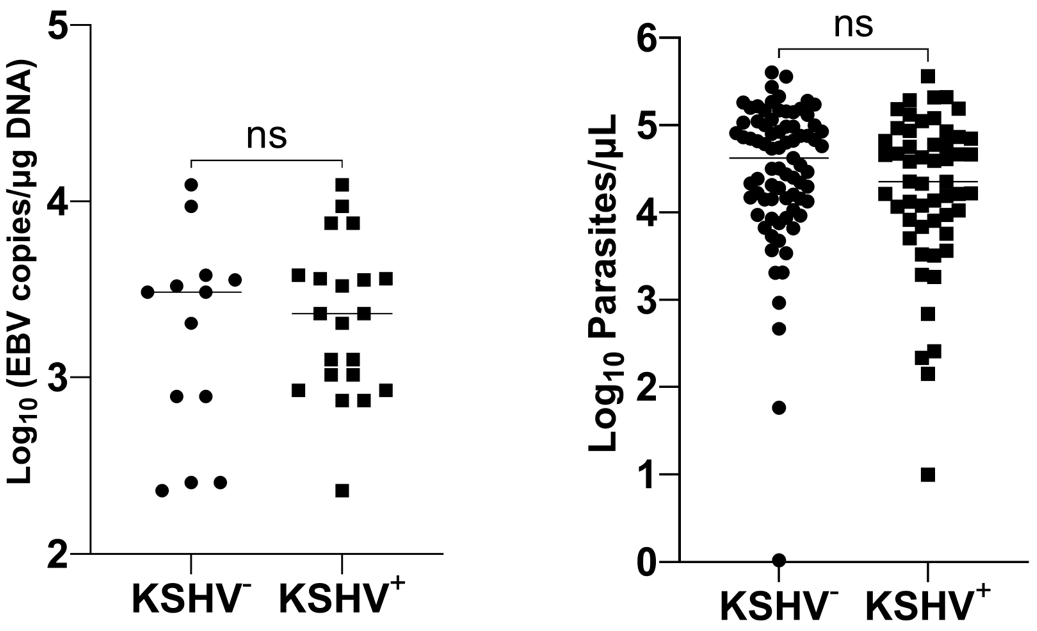

Figure S3. EBV and malaria parasite density in KSHV seropositive and seronegative A-KSM children.
